# Vitamin A Enhances Antitumor Effect of a Green Tea Polyphenol on Melanoma by Upregulating the Polyphenol Sensing Molecule 67-kDa Laminin Receptor

**DOI:** 10.1371/journal.pone.0011051

**Published:** 2010-06-10

**Authors:** Ju Hye Lee, Mutsumi Kishikawa, Motofumi Kumazoe, Koji Yamada, Hirofumi Tachibana

**Affiliations:** 1 Department of Bioscience and Biotechnology, Faculty of Agriculture, Kyushu University, Hakozaki, Japan; 2 Laboratory of Functional Food Design, Department of Functional Metabolic Design, Bio-Architecture Center, Kyushu University, Fukuoka, Japan; Mizoram University, India

## Abstract

**Background:**

Green tea consumption has been shown to have cancer preventive qualities. Among the constituents of green tea, (-)-Epigallocatechin-3-*O*-gallate (EGCG) is the most effective at inhibiting carcinogenesis. However, the concentrations of EGCG that are required to elicit the anticancer effects in a variety of cancer cell types are much higher than the peak plasma concentration that occurs after drinking an equivalent of 2–3 cups of green tea. To obtain the anticancer effects of EGCG when consumed at a reasonable concentration in daily life, we investigated the combination effect of EGCG and food ingredient that may enhance the anticancer activity of EGCG on subcutaneous tumor growth in C57BL/6N mice challenged with B16 melanoma cells.

**Methodology/Principal Findings:**

All-*trans*-retinoic acid (ATRA) enhanced the expression of the 67-kDa laminin receptor (67LR) and increased EGCG-induced cell growth inhibition in B16 melanoma cells. The cell growth inhibition seen with the combined EGCG and ATRA treatment was abolished by treatment with an anti-67LR antibody. In addition, the combined EGCG and ATRA treatment significantly suppressed the melanoma tumor growth in mice. Expression of 67LR in the tumor increased upon oral administration of ATRA or a combined treatment of EGCG and ATRA treatment. Furthermore, RNAi-mediated silencing of the retinoic acid receptor (RAR) α attenuated the ATRA-induced enhancement of 67LR expression in the melanoma cells. An RAR agonist enhanced the expression levels of 67LR and increased EGCG-induced cell growth inhibition.

**Conclusions/Significance:**

Our findings provide a molecular basis for the combination effect seen with dietary components, and indicate that ATRA may be a beneficial food component for cancer prevention when combined with EGCG.

## Introduction

Tea (*Camellia sinensis* L.) is one of the most widely consumed beverages in the world. (-)-Epigallocatechin-3-*O*-gallate (EGCG), which is the major green tea catechin present in the leaves, is believed to the compound most responsible for the health benefits attributed to tea. EGCG was reported to have antioxidative [1], [2], antimutagenic [3], anti-inflammatory [4], and anticarcinogenic activities [5].

Although the EGCG concentrations required to elicit the anticancer activity have been shown to be more than 1 µM, the blood level of EGCG after consuming the equivalent of 2–3 cups of green tea was 0.1–0.6 µM and for an equivalent of 7–9 cups was still lower than 1 µM [6], [7]. In a cohort study, daily consumption of ten cups of green tea was required for the cancer preventive effect [8]. Moreover, adverse effects of green tea, mainly hepatitis, by consumption of high doses of green tea have been reported [9]. Therefore, it is important to enhance the pharmacologic effect of EGCG to obtain the health benefit in reasonable concentration in daily life.

We have reported that the cell-surface binding of EGCG and its derivatives is involved in their biological activities [10]–[15]. We have identified the 67-kDa laminin receptor (67LR) as a cell surface receptor for EGCG that mediates the anticancer activity of EGCG [16]. 67LR has been shown to be overexpressed on the cell surface of various tumor cells [17]. It was postulated that 67LR plays a significant role in the tumor progression and speculated that studies conducted to define the function of 67LR could provide a new approach to cancer prevention. Indeed, expression of 67 LR confers EGCG responsiveness to tumor cells *in vivo* [18].

Vitamin A, also known as retinol, participates in physiological activities related to the immune system, maintenance of epithelial and mucosa tissues, growth, reproduction, and bone development. It comes from animal sources, such as eggs, meat, milk, cheese, cream, liver, kidney, cod and halibut fish oil. *In vitro* and in animal models, it has been demonstrated that vitamin A is involved in the regulation and promotion of growth and differentiation of many cells [19]. The visual function of vitamin A depends on its natural and synthetic derivatives, retinoids [20]. All-*trans*-retinoic acid (ATRA), the active derivative of vitamin A, has been well documented as a growth and differentiation factor in many tissues and cells, and proved to be an effective treatment to many diseases including cancers [21], [22].

Retinoids exert their physiological activities through retinoid receptor nuclear proteins that belong to the superfamily of steroid/thyroid hormone receptors, of which there are two classes, retinoic acid receptors (RARs) and the retinoic-X receptors (RXRs), each of which has three subtypes, α, β, and γ [23], [24]. The natural ligands for the RARs are ATRA and its stereoisomers 9-cis-RA and 13-cis-RA, whereas RXRs are activated by 9-cis-RA only. ATRA acts through RAR to transcriptionally activate target genes, such as cytochrome P450 and CRABI [24].

This study was designed to identify a food component that could be effectively used in combination with EGCG and to investigate the mechanism of action of this combination. By using *in vitro* and *in vivo* systems involving a highly metastatic mouse B16 melanoma cell line [25], we found that ATRA enhances the antitumor activity of EGCG by upregulating the 67 LR expression through RAR.

## Results

### ATRA enhances the 67LR expression and EGCG-induced cell growth inhibition in B16 cells

**Figure 1:**
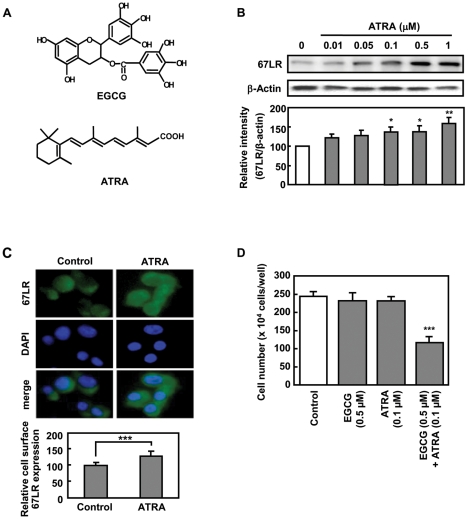
ATRA enhances the expression of 67LR and cell growth inhibitory activity of EGCG. **A**) Structure of EGCG and ATRA. **B**) 67LR protein levels in B16 cells exposed to the indicated concentrations of ATRA for 48 h were analyzed by Western blot analysis. Levels of 67LR expression were detected with anti 67LR serum, and were normalized to β-Actin. Band intensities were quantified using NIH Image J software. **C**) Anti-67LR antibody conjugated with Alexa Fluor 488 (1 mg/ml) was used at a dilution of 1∶100. Photographs were taken under Keyence BZ-8001 fluorescence microscope. **D**) Cells were counted after treatment with or without 0.5 µM EGCG and/or 0.1 µM ATRA in DMEM supplemented with 1% FCS for 48 h and 96 h each. Cell proliferation was evaluated by counting the number of cells using a Counlter Counter. Data shown are means ± S.D. for three samples. Data containing asterisk marks are significantly different from the values in control at ***p<0.001.

We previously reported that ATRA enhances the expression of 67LR on MCF-7 cells [16]. To determine whether ATRA enhances anti-tumor effect of EGCG *in vivo* model, we examined the 67LR expression on B16 melanoma cells by using Western blot analysis after treatment with different concentrations of ATRA. ATRA enhanced the expression of 67LR in a dose dependent manner (Fig. 1A). We also found that ATRA treatment increased the cell surface expression of the 67LR as compared with the expression in the control cells (Fig. 1B).

We next examined the effects of combined EGCG and ATRA treatment on cell growth of B16 cells. Combination treatment with ATRA (0.1 µM) and EGCG at a physiological concentration (0.5 µM) significantly suppressed the number of B16 cells to 52.4% of the control, whereas treatment with EGCG or ATRA alone did not inhibit cell growth (Fig. 1C). These results suggest that ATRA enhances EGCG-induced cell growth inhibition through 67LR upregulation in B16 cells.

### ATRA induced the cell growth inhibitory activity of EGCG through the enhancing of EGCG binding to 67LR

**Figure 2:**
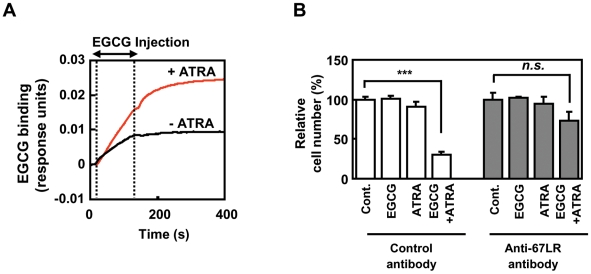
ATRA induces the EGCG activity through the enhancing of EGCG binding to 67LR. **A**) EGCG binding to the surface of B16 cells treated with (red line) or without (black line) ATRA monitored by surface plasmon resonance. EGCG was injected at a concentration of 5 µM for the indicated time interval (+ EGCG). **B**) B16 cells treated with 0.1 µM ATRA in DMEM supplemented with 1% FCS for 48 h. Then, the cells were treated with either anti-67LR (MLuC5) or control antibody (mouse IgM) for 2 h and then the cells were added to 0.5 µM EGCG for 48 h. Cell proliferation was assessed by the WST-1 reagent. Cell number was measured as 430 nm absorbance and shown as relative of control. Data shown are means ± S.D. for three samples. Data containing asterisk marks are significantly different from the values in control at ***p<0.001.

Cell surface binding of EGCG was assessed using SPR biosensor assay (Fig. 2A). We found that ATRA significantly enhances the binding of EGCG to cells surface of B16 cells. To investigate the participation of 67LR in ATRA-induced the cell growth inhibitory activity of EGCG, B16 cells were treated with an anti-67LR antibody. The growth of the cells treated with a control antibody was inhibited by the combined EGCG and ATRA treatment (Fig. 2B). This growth-suppressive effect was eliminated upon treatment with an anti-67LR antibody. Together, these observations show that ATRA action for the cell growth inhibitory activity of EGCG is attributable to the enhancement of cell surface binding of EGCG via 67LR.

### Combined EGCG and ATRA treatment suppresses tumor growth *in vivo*

**Figure 3:**
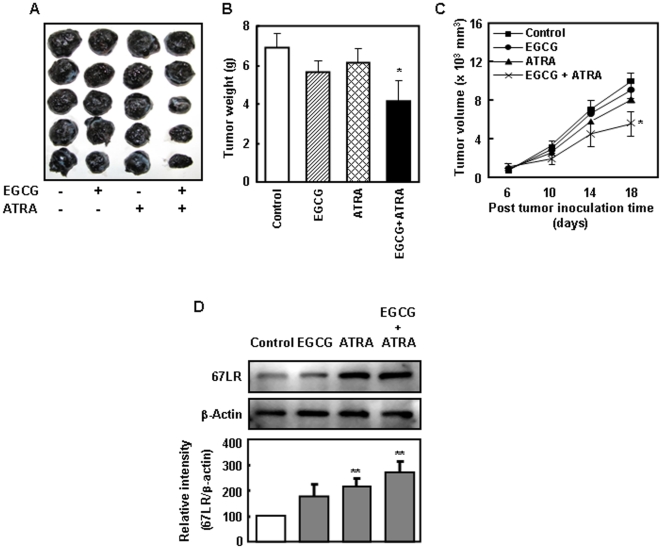
Tumor growth and expression levels of 67LR after the treatment ATRA and EGCG *in vivo*. C57BL/6N mice were subcutaneosly inoculated with B16 cells. The administration of 0.1% EGCG, ATRA (10.5 mg/kg), and combination of EGCG and ATRA was started 1 day before the cell inoculation. **A**) We excised tumors from mice 22 days after cell inoculation and photographed them. **B**) Tumor weights are represented as the mean ± S.E. of five mice. Logarithmic transformation for them were analyzed using an one-way ANOVA coupled with a Dunnet test in which *p<0.05 was the minimum requirement for a statistically significant difference from control group. **C**) Tumor volumes was measured in two dimensions and calculated as follows: lengh/2× width^2^. Each data point represents the mean ± S.E. of tumor volumes from five animals and they are significantly different from control group at *p<0.05 (Mann-Whitney *U* test). **D**) Representative Western blot analyses of 67LR from each individual mouse. Levels of these proteins expression were normalized to β-Actin. Band intensities were quantified using NIH Image J software.

To determine the *in vivo* efficacy and safety of the combined treatment, mice were implanted with B16 cells and treated with EGCG and/or ATRA. Compared to treatment with a vehicle control, combined treatment significantly reduced the tumor volume over the duration of the study (Fig. 3A, B, and C). The tumor volume and weight in mice treated with EGCG or ATRA alone did not differ from those in mice treated with the vehicle control. On the other hand, the mean tumor weight in the combination-treatment group was ∼40% less than that in the control group, indicating that ATRA intensifies the anti-tumor activity of EGCG. Mice subjected to the combination treatment lost 0.6 g of weight (data not shown). All other physiological parameters (*i.e*., liver, kidney, spleen, and uterine weight) did not show any other obvious side effects.

To examine whether 67LR are involved in the inhibition of tumor growth, we measured the expression of 67LR in the tumor cells by using Western blot analysis. As shown in Fig. 3D, the 67LR levels in the tumor were increased upon oral administration ATRA, or combination of EGCG and ATRA. These results suggest that ATRA enhances the EGCG-induced inhibition of tumor growth through 67LR upregulation *in vivo*.

### Enhancement of 67LR expression by ATRA is mediated through RARα

**Figure 4:**
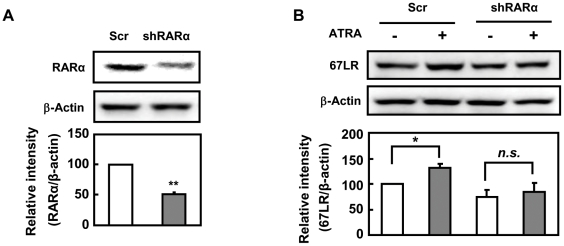
ATRA enhances the 67LR expression via RARα. **A**) RARα knockdown in B16 cells stably transfected with RARα shRNA expression vector was confirmed by Western blot analysis. **B**) B16 cells stably transfected with the control shRNA or the RARα shRNA expression vector were treated with or without 0.1 µM ATRA in DMEM supplemented with 1% FCS for 48 h. Levels of 67LR expression were analyzed by Western blot analysis and were normalized to β-Actin. Band intensities were quantified using NIH Image J software.

RAR that binds to ligand ATRA form a heterodimer with RXRs and regulate the expression of specific genes [26], [27]. To investigate whether the ATRA-induced enhancement of 67LR expression is mediated through RARα, B16 cells were stably transfected with RARα shRNA expression vector that allows knockdown of RARα (Fig. 4A). Knockdown of RARα attenuated the ATRA-induced enhancement of 67LR expression (Fig. 4B). These results suggest that ATRA enhances 67LR expression through RARα.

### RAR agonist enhances EGCG-induced cell growth inhibition through 67LR upregulation

**Figure 5:**
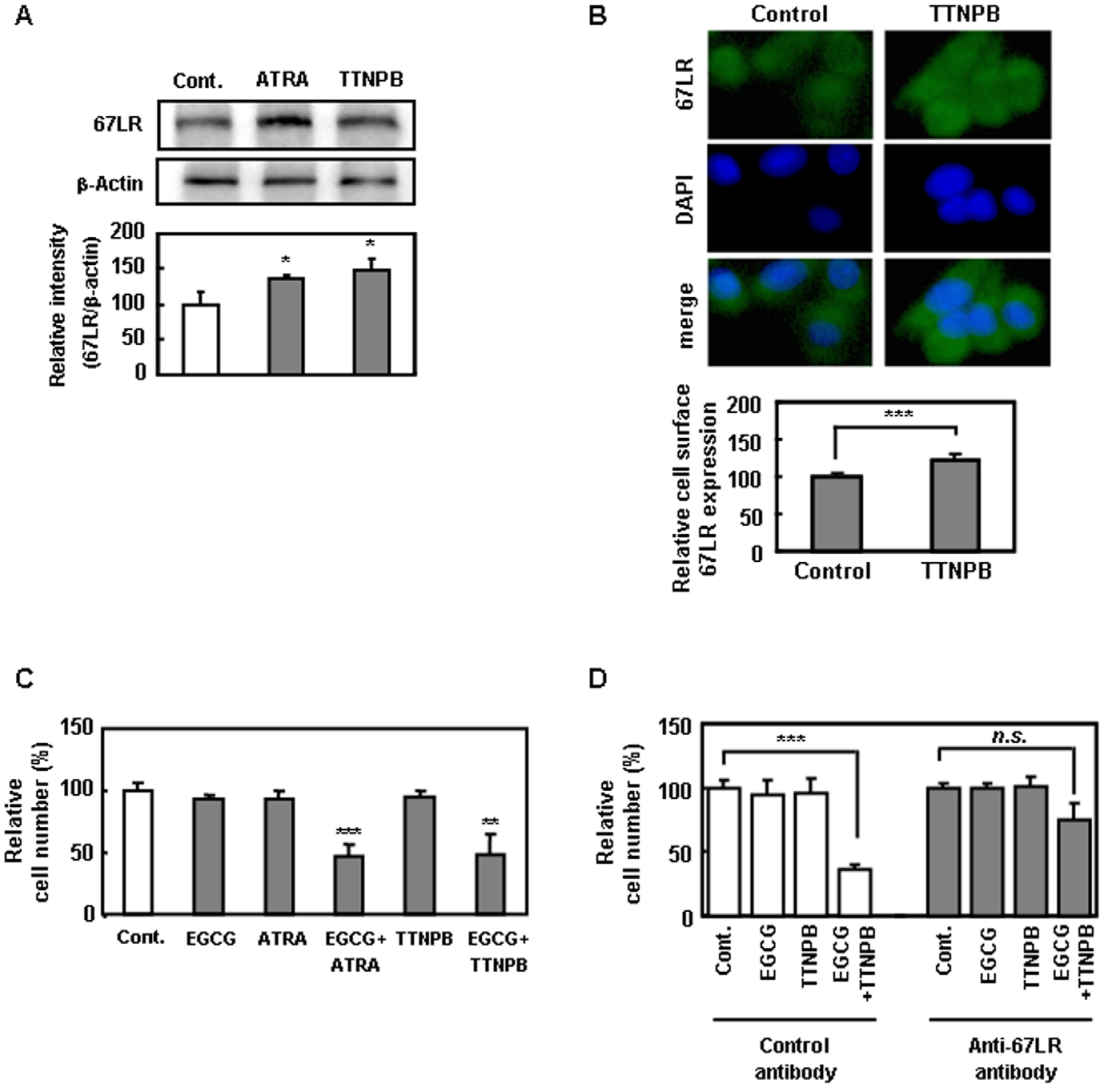
RAR agonist induced EGCG-elicited cell growth inhibition through 67LR upregulation. **A**) Cells were treated with or without 0.1 µM ATRA or 0.1 µM TTNPB in DMEM supplemented with 1% FCS for 48 h and were analyzed by Western blot analysis. Levels of 67LR expression were normalized to β-Actin. Band intensities were quantified using NIH Image J software. **B**) Anti-67 LR antibody conjugated with Alexa Fluor 488 was used at a dilution of 1∶100. Photographs were taken under Keyence BZ-8100 fluorescence microscope. **C**) Cells were treated with 0.1 µM ATRA or RARα agonist, 0.1 µM TTNPB in DMEM supplemented with 1% FCS for 48 h, then treated with 0.5 µM of EGCG for 48 h. Data shown are means ± S.D. for three samples. Data containing asterisk marks are significantly different from the values in control at **p<0.01, ***p<0.001. **D**) B16 cells were treated with 0.1 µM TTNPB in DMEM supplemented with 1% FCS for 48 h, then the cells were treated with either anti-67LR (MLuC5) or control antibody (mouse IgM) for 2 h, and the cells were added 0.5 µM of EGCG for 48 h. Cells proliferation was assessed by the WST-1 reagent. Cell number was measured as 430 nm absorbance and shown as relative of control. Data shown are means ± S.D. for three samples. Data containing asterisk marks are significantly different from the values in control at ***p<0.001.

To investigate the participation of RAR in ATRA-mediated enhancement of EGCG-induced cell growth inhibition through 67LR, B16 cells were treated with the pan-RAR agonist TTNPB. TTNPB enhanced the protein levels and cell-surface levels of 67LR after 48 h of treatment (Fig. 5A, B). Moreover, treatment with TTNPB enhanced EGCG-induced cell growth inhibition (Fig. 5C). The growth-suppressive effect by combination treatment with EGCG and TTNPB was obviously reduced upon treatment with anti-67LR antibody (Fig. 5D). These results suggest that RAR-mediated action is involved in the ATRA-induced enhancement of EGCG-elicited cell growth inhibition.

## Discussion

Recent studies have suggested a causal association between high doses of green tea-containing dietary supplements and liver damage [9]. Laboratory study of green tea-derived preparations in rodents has also shown revealed toxic effects at high doses [28]. In human, hepatotoxicity was reported in a 45-year-old man following consumption of 6 cups of green tea per day for 4 months [29]. To determine the anticancer effects of EGCG at the concentration (0.5 µM) found in human plasma after consuming two or three cups of green tea [6], we studied the ideal combination of EGCG and food ingredient that may enhance the antitumor activity of EGCG *in vivo*. Here, we showed that ATRA enhanced EGCG-induced tumor growth inhibition by upregulatiing of the 67LR *in vivo*.

ATRA has been used as chemopreventive and therapeutic agents for the treatment of a wide variety of tumors [30], [31], [32], especially in combination with another therapeutic agent [33]. Huang et al. have reported that ATRA intensifies rosiglitazone-induced growth inhibition and differentiation in multiple myeloma cells [34]. In addtion, Karmakar et al. have shown that the combination of ATRA and paclitaxel induces cell differentiation and apoptosis in human glioblastoma U87MG xenografts in nude mice [35]. Therefore, ATRA may be a potent candidate for an effective EGCG-based therapeutic combination for cancer prevention. This hypothesis is supported by our previous report showing that ATRA enhances the binding of EGCG to the surface of cancer cells [16]. In this study, we showed that a combination treatment of EGCG and ATRA suppresses tumor growth in mice without causing any obvious side effects.

When subjects were given a normal dose of ATRA (45 mg/m^2^) that is commonly used as therapy for leukemia, the maximum plasma concentration of ATRA was approximately 1 µM [36]. The concentration of ATRA (0.1 µM) used in our study was much lower than the maximum concentration in human plasma. In consideration of these facts, the activities observed at 0.1 µM ATRA are relevant to an *in vivo* situation.

Although overexpression of 67LR has been correlated with increased aggressiveness and malignancy of some tumors [17], [37], [38], our results show that the enhancement of 67LR expression at the cell surface of melanoma cells is not associated with the incidence and volume of tumor. These results suggest that upregulation of 67LR alone does not correlate with the malignancy of melanoma.

Recently, we have shown new insights into the 67LR signaling pathway [18]. Our previous study showed that EGCG induces dephosphorylation of MYPT1 at Thr696 and activates myosin phosphatase through 67LR. In addition, EGCG-induced tumor growth inhibition was abrogated by silencing of 67LR, eEF1A, or MYPT1 in tumor cells, suggesting that the signaling pathway mediated by 67LR, eEF1A, and MYPT1 is indispensable for the anticancer effect of EGCG. These findings are implicated in that the 67LR signaling pathway may be involved in the combination of EGCG and ATRA-induced tumor growth inhibition.

This is the first report showing that 67LR expression is regulated by RAR. The finding is supported by a previous report showing that 37LRP, the precursor of 67LR, is upregulated in response to RA [39]. In contrast, RA has been shown to have an anticancer effect on leukaemic cells [40], despite the finding that RA reduces expression of 67LR in leukaemic cells [41]. Therefore, the effect of RA on the expression of 67LR expression is markedly depending on the cell type. It has been shown that such opposing effects of RA are affected by the expression of transcription factors. For example, RA activates the nuclear receptor PPARβ/γ in addition to RAR. The differentia partitioning of RA between the two receptors is regulated by the intracellular lipid binding proteins CRABP-II and FABP5. In cells that express a high CRABP-II/FABP5 ratio, RA is channeled to the RAR, which often results in growth inhibition. Conversely, in the presence of a low CRABP-II/FABP5 expression ratio, RA is targeted to PPARβ/γ, thereby upregulating survival pathways [42].

Our results showed that ATRA enhances 67LR expression through RARα, thus indicating that RARα is involved in ATRA-induced EGCG-mediated inhibition of cell growth inhibition via 67LR. Moreover, we showed that RAR agonist TTNPB enhances the protein levels and cell-surface levels of 67LR, and EGCG-induced cell growth inhibition. These indicate that any compounds which activate RAR may be a candidate to enhance the antitumor activity of EGCG.

The results shown in this study provide a molecular basis for the combination effect of dietary components. More definitive information on the cancer-preventive activity of combined EGCG and ATRA ingestion will emerge from cohort studies and human intervention trials.

## Materials and Methods

### Ethics Statement

All animal works were carried out in accordance with the law (number 105) and notification (number 6) of the Japanese government for the welfare of experimental animal. All procedures were approved by the Animal Care and Use Committee of Kyushu University.

### Materials and Antibodies

EGCG was purchased from Sigma and DSM Nutritional Products, Inc. (Parsippany, NJ). Catalase and anti-β-Actin antibody were purchased from Sigma. Anti-67LR antibody (MLuC5) was purchased from Abcam (ab80582) and anti-67LR serum was obtained from the rabbit which was immunized with synthesized peptide corresponding to residues 161–170 of human 67LR. Anti-RARα antibody was purchased from Santa Cruz Biotechnology, Inc. (Santa Cruz, CA). ATRA was purchased from Sigma and was prepared as 10 mM stocks in 100% ethanol. ((E)-4-[2-(5,6,7,8-tetrahydro-5,5,8,8-tetramethyl-2-naphthylenyl)-1-propenyl] benzoic acid (TTNPB) were purchased from Sigma-Aldrich and was dissolved in ethanol at 10 mM. These stocks were diluted with the media to the desired concentrations immediately before each experiment, keeping the final concentration of ethanol at 0.1%. All experiments were performed under low-light conditions to minimize retinoid photoisomerization.

### Cell Culture and RNA interference by short hairpin RNA (shRNA)

Mouse melanoma B16 cells were maintained in Dulbecco's modified Eagle's medium containing 5% FCS. To assess cell proliferation, cells were treated with EGCG or ATRA for 48 h in Dulbecco's modified Eagle's medium supplemented with 1% FCS, 5 mg/ml BSA, and 200 units/ml catalase. FuGene6 transfection reagent (Roche Applied Science) was used for stable transfection of cells, according to the manufacturer's protocol. For selecting stable clones, transfected cells were grown in medium containing G418 for neomycin resistance.

### Anti-67LR antibody treatment for Proliferation assay

Cell proliferation was assessed by the water-soluble tetrazolium salt (WST-1) reagent according to the manufacturer's instructions (Roche Molecular Biochemicals, Germany). B16 cells were treated with ATRA or TTNPB in DMEM supplemented with 1% FCS for 48 h. Then, the cells were treated with either anti-67LR (MLuC5) or control antibody (mouse IgM) for 2 h and the cells were cultured in the medium containing 0.5 µM EGCG for 48 h. The absorbance of each well was measured at 430 nm with a microplate reader (LS-PLATE manager 2001, Wako).

### Western Blot Analysis

Cells were lysed in cell lysis buffer containing 50 mM Tris-HCl (pH 7.5), 150 mM NaCl, 1% Triton X-100, 1 mM EDTA, 50 mM NaF, 30 mM Na_4_P_2_O_7_, 1 mM phenylmethanesulfonyl fluoride, 2 µg/ml aprotinin, and 1 mM pervanadate. Proteins were resolved on SDS-polyacrylamide gels and then transferred onto a nitrocellulose membrane. The membranes were blocked in 2.5% BSA and incubated with the antibody, followed by incubation with a secondary antibody. Proteins were visualized by using the ECL Advance kit (Amersham Biosciences). Band intensities were quantified using NIH Image-J software.

### Binding analysis using surface plasmon resonance biosensor

B16 cells were cultured with or without 1 µM ATRA for 24 h, and were subjected to EGCG binding assay using the surface plasmon resonance (SPR) biosensor SPR670 (Moritex Corp., Tokyo, Japan). The cells were immobilized on a dithiodibutyric acid-coated sensor chip, after activation with carbodiimide (16 mM) and *N*-hydroxysuccinimide (13 mM). Phosphate-buffered saline (PBS, pH 7.4) was used as a mobile phase medium at a flow rate of 30 µl/min at 25°C. Binding of EGCG (1 µM) to the immobilized cells was monitored in real time by measuring changes in resonance unit. The sensorgrams for the reference channel (sample-free PBS) were subtracted simultaneously from the sensorgrams for sensing channel (sample-containing PBS) on the same sensor chip.

### Fluorescent Microscope

Cells were fixed 4% paraformaldehyde in PBS pH 7.4 for 30 min on ice. And then cells were incubated with 3% BSA in PBS for 2 h. After this blocking, Cells were incubated with 10 µg/mL of the anti-67LR antibody conjugated with Alexa Fluor 488 for 2 hours on ice. After washing, cells were observed under the fluorescent microscopy (KEYENCE BZ-8100).

### Tumor Growth *in vivo*

B16 cells were detached and resuspended in phosphate-buffered saline. 5×10^5^ cells in a single cell suspension were injected subcutaneously into the back of C57BL/6N mice (Charles River Laboratories Japan, Yokohama, Japan). They were kept at the Biotron Institute of Kyushu University in a 12-h light/12-h dark cycle (light on at 8 a.m.) in an air-conditioned room (20°C and 60% humidity under specific pathogen-free conditions). The mice randomly assigned to 4 groups for treatment. Mice were treated with EGCG, ATRA, EGCG+ATRA, or a vehicle control for 23 days. EGCG was dissolved in vehicle (0.01% ascorbic acid solutions (pH 5.5) adjusted by NaOH). One day before inoculation, drinking water bottles were replaced by 0.1% EGCG every 2 days. ATRA was dissolved in vehicle (olive oil) and administered through oral gavage every 3 days. Dosing solutions were prepared fresh each day. Tumor sizes were determined every other day via caliper measurements. The tumor volume was measured in two dimensions and calculated as follows: length/2× width^2^. Each data point represents the mean ± S.E. of tumor volumes from 6–7 animals.

### Statistical Analysis

Data for tumor growth in vivo were analyzed by a Mann-Whitney U test. The other data were analyzed by Student's t test. A level of p<0.05 was considered significant.
